# *Treponema pallidum* genetic diversity and its implications for targeted vaccine development: A cross-sectional study of early syphilis cases in Southwestern Colombia

**DOI:** 10.1371/journal.pone.0307600

**Published:** 2024-07-19

**Authors:** Juan C. Salazar, Fabio Vargas-Cely, Jonny A. García-Luna, Lady G. Ramirez, Everton B. Bettin, Nelson Romero-Rosas, María F. Amórtegui, Sebastián Silva, Oscar Oviedo, Julie Vigil, Carson J. La Vake, Ximena Galindo, Jose D. Ramirez, Alvaro J. Martínez-Valencia, Melissa J. Caimano, Christopher M. Hennelly, Farhang Aghakhanian, M. Anthony Moody, Arlene C. Seña, Jonathan B. Parr, Kelly L. Hawley, Eduardo López-Medina, Justin D. Radolf

**Affiliations:** 1 Department of Pediatrics, University of Connecticut School of Medicine, Farmington, CT, United States of America; 2 Department of Immunology, University of Connecticut School of Medicine, Farmington, CT, United States of America; 3 Division of Infectious Diseases, Connecticut Children’s, Hartford, CT, United States of America; 4 Centro Internacional de Entrenamiento e Investigaciones Médicas (CIDEIM), Cali, Colombia; 5 Universidad ICESI, Cali, Colombia; 6 Division of Dermatology, School of Medicine, Universidad del Valle, Cali, Colombia; 7 Department of Medicine, University of Connecticut School of Medicine, Farmington, CT, United States of America; 8 Corporación de Lucha Contra el SIDA, Cali, Colombia; 9 Department of Molecular Biology and Biophysics, UConn Health, Farmington, CT, United States of America; 10 Institute for Global Health and Infectious Diseases, University of North Carolina, Chapel Hill, NC, United States of America; 11 Duke Human Vaccine Institute, Durham, NC, United States of America; 12 Department of Pediatrics, Duke University Medical Center, Durham, NC, United States of America; 13 Department of Integrative Immunology, Duke University Medical Center, Durham, NC, United States of America; 14 Centro de Estudios en Infectología Pediátrica (CEIP), Cali, Colombia; 15 Department of Genetics and Genome Sciences, UConn Health, Farmington, CT, United States of America; Food and Drug Administration, UNITED STATES OF AMERICA

## Abstract

**Background:**

Venereal syphilis, caused by the spirochete *Treponema pallidum* subsp. *pallidum* (*TPA*), is surging worldwide, underscoring the need for a vaccine with global efficacy. Vaccine development requires an understanding of syphilis epidemiology and clinical presentation as well as genomic characterization of *TPA* strains circulating within at-risk populations. The aim of this study was to describe the clinical, demographic, and molecular features of early syphilis cases in Cali, Colombia.

**Methods and findings:**

We conducted a cross-sectional study to identify individuals with early syphilis (ES) in Cali, Colombia through a city-wide network of public health centers, private sector HIV clinics and laboratory databases from public health institutions. Whole blood (WB), skin biopsies (SB), and genital and oral lesion swabs were obtained for measurement of treponemal burdens by *polA* quantitative polymerase chain reaction (qPCR) and for whole-genome sequencing (WGS). Among 1,966 individuals screened, 128 participants met enrollment criteria: 112 (87%) with secondary (SS), 15 (12%) with primary (PS) and one with early latent syphilis; 66/128 (52%) self-reported as heterosexual, while 48 (38%) were men who have sex with men (MSM). Genital ulcer swabs had the highest *polA* copy numbers (67 copies/μl) by qPCR with a positivity rate (PR) of 73%, while SS lesions had 42 *polA* copies/μl with PR of 62%. WB *polA* positivity was more frequent in SS than PS (42% vs 7%, respectively; p = 0.009). Isolation of *TPA* from WB by rabbit infectivity testing (RIT) was achieved in 5 (56%) of 9 ES WB samples tested. WGS from 33 Cali patient samples, along with 10 other genomic sequences from South America (9 from Peru, 1 from Argentina) used as comparators, confirmed that SS14 was the predominant clade, and that half of all samples had mutations associated with macrolide (*i*.*e*., azithromycin) resistance. Variability in the outer membrane protein (OMP) and vaccine candidate BamA (TP0326) was mapped onto the protein’s predicted structure from AlphaFold. Despite the presence of mutations in several extracellular loops (ECLs), ECL4, an immunodominant loop and proven opsonic target, was highly conserved in this group of Colombian and South American *TPA* isolates.

**Conclusions:**

This study offers new insights into the sociodemographic and clinical features of venereal syphilis in a highly endemic area of Colombia and illustrates how genomic sequencing of regionally prevalent *TPA* strains can inform vaccine development.

## Introduction

Venereal syphilis, a chronic sexually transmitted infection (STI) caused by the spirochete *Treponema pallidum* subspecies *pallidum* (*TPA*), continues to be a major global public health crisis [1]. The World Health Organization (WHO) estimates that seven million people are infected annually [2] with the Americas contributing three million new cases every year [3]. Persistently high rates of venereal syphilis in the general population, combined with a lack of proper access to adequate prenatal care, are responsible for the dramatic resurgence of gestational (GS) and congenital (CS) syphilis [4–9]. Mother-to-child transmission of the bacterium leads to devastating adverse birth outcomes, such as prematurity, stillbirth, and neonatal death [10, 11] and is responsible for over 200,000 perinatal deaths per year [4, 5]. In Colombia, mandatory reporting for venereal syphilis is not required by public health authorities; consequently, the actual incidence and prevalence of the disease in the general population is not well established. Nonetheless, published reports suggest syphilis is widespread, particularly in high-risk groups, such as commercial sex workers, men who have sex with men (MSM) and people living with HIV (PLWH) [12–16]. Mandatory reporting, on the other hand, is required for all GS and CS cases in the country. In 2021, 10,117 new cases of GS were reported in Colombia, representing a 155% increase compared to 2015 [17]. Likewise, a 181% increase in cases of CS, from 1.1 to 3.1 cases per 1,000 live births, occurred over the same period. These epidemiologic trends underscore the critical need for enhanced public health strategies to curtail syphilis transmission in this Latin American country along with the urgency of developing a safe and effective vaccine with regional as well as global coverage.

To develop and deploy a syphilis vaccine that will be effective globally, it is important to understand local risk factors associated with acquisition and transmission of the disease, barriers to timely diagnosis and care, typical and atypical clinical presentations, and, most importantly, the genomic diversity of circulating *TPA* strains. Defining the molecular epidemiology of *TPA* is complicated by the inability to culture the bacterium directly from clinical samples. To overcome these limitations, we and others have used molecular methodologies to assess *TPA* burdens in specimens from persons with well characterized early syphilis (ES) [18–20]. Whole genome sequencing (WGS) of *TPA* from clinical samples now allows us to establish the geographic and temporal distribution of circulating *TPA* clades and subtypes. With few exceptions, most *TPA* genomes originated from the USA [21], Western Europe [22] and China [23]. Our understanding of the epidemiology and genomic diversity of *TPA* is scarce in many regions of the world, including Latin America, where the disease is rampant [24].

Herein, as part of a multicenter global consortium for vaccine development [25], we analyzed the sociodemographic, clinical, microbiologic, and molecular features of ES patients enrolled longitudinally over an eight-year period in Cali, Colombia. Our findings confirm that venereal syphilis is a markedly underrecognized and underreported public health problem in this region of Colombia. WGS revealed that, as observed elsewhere [23, 25, 26], SS14-lineage *TPA* strains predominate over strains belonging to the Nichols clade. Our recent characterization of the bacterium’s outer membrane protein (OMP) repertoire [27, 28] provides a road map to distinguish globally conserved *TPA* surface antigens that could be used for the development of a syphilis vaccine. We used genomic sequences to examine the variability of BamA/TP0326, a promising candidate vaccinogen [27, 29, 30]. Despite the presence of mutations in several extracellular loops (ECLs) of BamA/TP0326, ECL4, an immunodominant loop and proven opsonic target [29, 31], was highly conserved in this group of Colombian and South American *TPA* strains. We found only one *TPA* strain exhibiting single-nucleotide polymorphisms (SNPs) in BamA ECL4 that give rise to nonconservative amino acid substitutions. This study offers new insights into the sociodemographic and clinical features of venereal syphilis in a highly endemic area of Colombia and illustrates how genomic sequencing can inform vaccine development.

## Materials and methods

This manuscript follows the Strengthening the reporting of observational studies in epidemiology–molecular epidemiology (STROBE-ME) guidelines.

### Study design

We carried out an observational, cross-sectional study of prospectively enrolled early syphilis (ES) cases between February 21^st^, 2014, and June 13^th^, 2022. Participants included in the period between February 21^st^, 2014, and November 18^th^, 2019, were prospectively recruited for other projects and their samples and data retrospectively included in this analysis (see ethics statement below). Participants recruited after November 18^th^, 2019, were prospectively included for this analysis.

### Subject identification and recruitment

**Fig 1:**
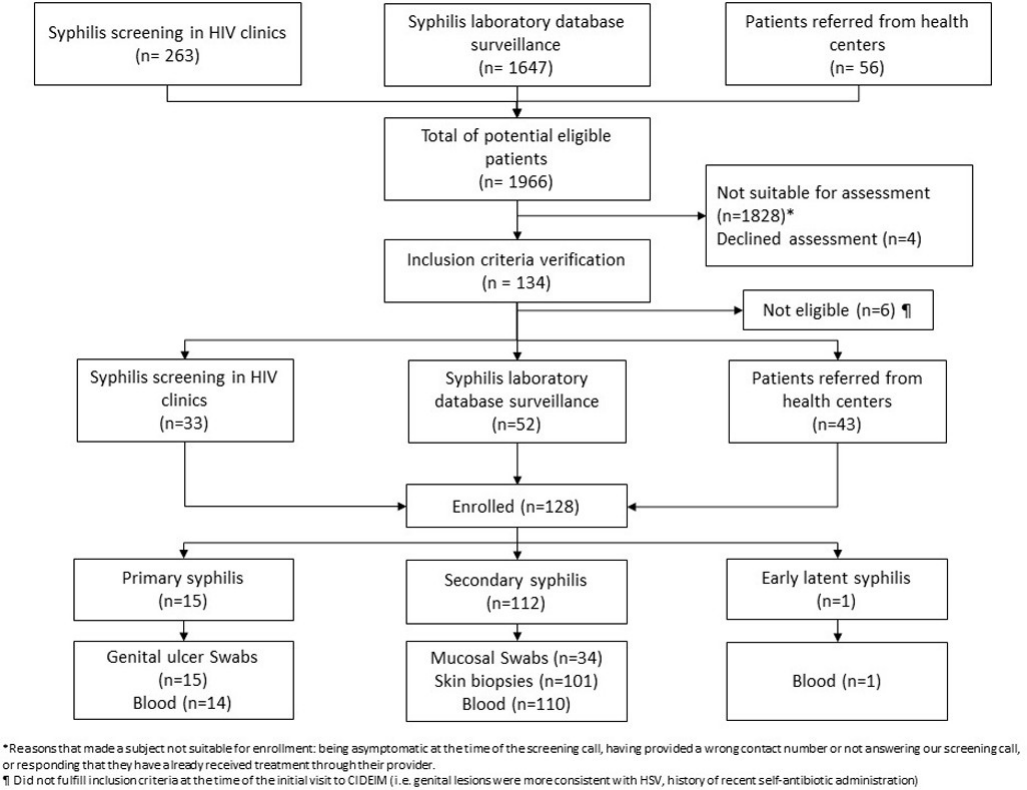
Screening and enrollment flowchart.

As depicted in **Fig 1**, ES patients were identified and referred to Centro Internacional de Educación e Investigaciones Médicas (CIDEIM) from three different sources: (i) In 2014, we established a public health outpatient clinic referral network consisting of 104 city wide, primary care institutions to enroll participants with ES. (ii) In 2019, we added two private HIV clinics to the network. (iii) In 2020, we initiated an active surveillance program using existing laboratory databases from public health institutions reporting individuals with reactive syphilis serologic tests, either treponemal tests (TT) or non-treponemal tests (NTT). For the latter, patients with reactive syphilis serologic tests were contacted via telephone and asked if they had signs or symptoms that could potentially fulfill one of our case definitions (see below). Individuals potentially fulfilling a case definition were invited to our clinical translational research center (CIDEIM) for evaluation by a trained physician who verified the diagnosis. Throughout the study period, our team routinely provided training in the recognition and management of ES to clinical staff at the public health centers which served as referral sites.

### Case definition and inclusion criteria

Potential study participants were 18 years or older, had not received antibiotics with activity against *TPA* in the last 30 days and fulfilled one of the following case definitions for ES, independently of their pregnancy or HIV status: Participants were considered to have primary syphilis (PS) if they had a characteristic ulcer or ulcers in the genital region, oral cavity, or perianal region along with a reactive NTT and/or confirmatory rapid TT. Participants with ulcers but non-reactive NTTs met criteria for PS if the ulcer exudate was positive by darkfield microscopy (DFM) and/or if *TPA* DNA was detected upon subsequent quantitative polymerase chain reaction (qPCR) analysis of the ulcer exudate as described below. A diagnosis of secondary syphilis (SS) was based on the presence of characteristic skin or mucosal lesions, a positive NTT (*i*.*e*., rapid plasma reagin ‐ RPR) and a positive confirmatory TT. An asymptomatic, untreated patient with a positive NTT and evidence of syphilis seroconversion within the 12 months prior to enrollment was considered to have early latent syphilis (ELS). Prior to 2019, persons living with HIV (PLWH), participants taking immunosuppressive medications and those with primary or ELS were not enrolled. Participants who ultimately met inclusion criteria in each of the three groups were invited to participate and included in the study following informed consent. Enrollment criteria, clinical history, physical examination findings and laboratory results for each patient were reviewed by two syphilis experts (JCS and EL) prior to assigning a definitive diagnosis of ES with staging.

### Study procedures

Following informed consent, clinical and sociodemographic data were collected employing a standard clinical record survey and double-entry paper-based case report form (CRF) for patients included between 2014 and 2018 or an electronic CRF (eCRF) in REDCap for patients enrolled between 2019 and 2022. Photographic images of lesions were filed with a predetermined anonymous codification to avoid linkage of the photographs with personal identifiers.

### Sample collection

Whole blood (WB) was collected for clinical and research purposes. Samples for clinical purposes were sent in temperature-controlled boxes to the reference laboratory of Fundación Valle del Lili (Cali), 5 mL in EDTA tubes for complete blood count, and 10 mL in serum separator tubes for hepatitis C antibodies, hepatitis B surface antigen and manual flocculation RPR. Additionally, a point of care rapid syphilis SD Bioline Syphilis 3.0 (Standard Diagnostics Inc. Kyonggi-do, Korea) was used as standard TT for all patients as we previously described [32]. A rapid HIV SD Bioline HIV 1/2 3.0 (Standard Diagnostics Inc. Kyonggi-do, Korea) was performed for HIV screening in all patients without a previous history of HIV infection.

Between 2014 and 2018 a single 4 mm punch skin biopsy was obtained from characteristic SS lesions. From 2019 onwards, two 4 mm punch skin biopsies were obtained, and two swabs were obtained from genital ulcers, mucous patches and/or condyloma lata. In PS patients, one of the genital ulcer swabs was used for DFM and the other for qPCR and WGS (see below). If a genital ulcer was assumed to have low treponemal burdens due to small size and/or obvious healing, swabs were prioritized for qPCR and WGS. Oral swabs, if present in PS patients, were also collected for qPCR and WGS. All biological specimens were transported from the clinic research site to the research laboratories using climate-controlled conditions and temperature monitory systems.

### Extraction and quantitation of *T. pallidum* DNA

#### DNA extraction from WB

A total volume of 2 ml of WB was obtained from all participants for DNA extraction within 2 hours of collection. DNA was extracted using the QIAmp DNA Blood Midi Kit according to the manufacturer’s instructions (Qiagen Inc., Valencia, CA) and DNAs were stored at -80°C until analysis. **DNA extraction from skin biopsies:** Skin samples collected between 2014 and 2018 were snap frozen and stored in cryopreservation. Alternatively, skin samples collected after 2018 were immediately placed in 500 μl of DNA/RNA Shield™ (Zymo Research Corporation) in a 1.8 ml cryogenic tube and stored at -80°C. Specimens were thawed and DNAs were extracted as previously described [14]. Briefly, skin biopsies were pre-treated with Type IV collagenase A (Gibco) at 37°C for at least 4 hrs followed by incubation with Proteinase K (20 mg/ml) overnight at 56°C prior to DNA extraction. **DNA extraction from genital ulcer swabs**: Following collection, swab samples were immediately placed in 250 μl of DNA/RNA Shield™ (Zymo Research Corporation). The shaft of each swab was cut off using sterile scissors and the applicator tip was sealed in a 1.8 ml cryogenic tube and stored at -80°C until the DNAs were extracted. Briefly, the swab applicator and the 250 μl of DNA/RNA Shield™ were treated with Proteinase K (20 mg/ml) for 4 hrs at 56°C within the original collection tube. DNA extraction was performed with the DNeasy blood and tissue kit (Qiagen Inc., Valencia, CA) according to the manufacturer’s protocol tissue kit. DNA obtained was quantified using a Nanodrop® ND-1000 and stored at -80°C.

#### *T. pallidum* DNA quantitation

PCR amplification of the *TPA polA* (*tp0105*) gene was performed as previously described [14] using Tp-polA-Forward 5’-CAGGATCCGGCATATGTCC-3 and Tp-polA-Reverse:5’-AAGTGTGAGCGTCTCATCATTCC-3’.FAM as primers and 5’CTGTCATGCACCAGCTTCGACGTCTT-3’-BHQ1 as the probe. Thermocycling was performed in CFX96 real-time PCR detection (CFX96; Bio-Rad, USA) as follows: 1 cycle of 95°C for 10 minutes followed by 40 cycles of 95°C for 15 seconds and 60°C for 1 minute. Each PCR run included positive and negative (no template) control reactions. *PolA* copy numbers were extrapolated from a standard curve generated using 10-fold serial dilutions (10^6^ to 10^1^ copies) of purified plasmid DNA containing the *polA* amplicon cloned into pCR2.1-TOPO vector (Invitrogen). The *polA* values obtained were then used as relative measurements to compare individual biological samples.

#### Rabbit infectivity testing (RIT)

The isolation of clinical strains of *TPA* utilizing the *in vitro* cultivation system have, to date, not been successful, therefore, RIT continues to be the “gold” standard to isolated clinical strains. Healthy adult male New Zealand white rabbits (O*ryctolagus cuniculus*) were obtained from the reproduction and breeding center of the animal facility of Universidad ICESI and used for isolation of clinical strains of *TPA* between 2020 and 2022. Rabbits were housed in a controlled temperature environment set at 12°C configured to house four rabbits in individual cubicles. Temperatures remained stable between 10°C and 12°C during 24 hr. reads. Prior to inoculation, rabbits were sedated by intramuscular injection with Tranquilan (maleate of acepromazine 1 mg/kg), and Xylazine (2 mg/kg) and then anesthetized with intravenous Zoletil 50 (tiletamine-zolazepam 10 mg/kg). Following inoculation, the analgesic flunixin meglumine (1.1 mg/kg) was administered intramuscularly. A total of 1.5 ml of WB obtained from ES patients was injected into each testis as previously described [33]. A weekly physical examination and venipuncture of the marginal ear vein were performed by a trained veterinarian. Animals were assessed to detect signs of orchitis, or systemic infection and serum was assessed for syphilis with rapid treponemal tests and by the RPR. For blood draws a restraining box was used to avoid further exposure to sedative medication and short nature of the procedure (< 2 min). Animals were euthanized under general anesthesia upon the development of orchitis, seroconversion, or at 90 days following inoculation without seroconversion. Typically, orchitis does not change animal behavior (*i*.*e*., alteration of movement patterns, feeding habits or water consumption). Animals were euthanized if they developed signs of sepsis or testicular necrosis. The humane euthanasia procedure involved the initial sedation of the animal using a cocktail of Tranquilan (acepromazine maleate) at a dosage of 1 mg/kg, along with Xilasyna 2 (Xylazine) administered at 2 mg/kg intramuscularly (IM). Subsequently, the rabbits were anesthetized using Zoletil 50 (tiletamine-zolazepam) at a dosage of 10 mg/kg intravenously (IV). To ensure the appropriate depth of anesthesia, the animals were closely monitored. Euthanasia was then carried out by injecting Euthanex (pentobarbital and sodium diphenylhydantoin) at a dosage of 1 ml/kg IV. The confirmation of the animal’s death was established by observing the absence of breathing and the cessation of a heartbeat. Spirochetes were recovered from rabbit testis and subsequently assessed by DFM. This was followed by determining the *TPA* burden through qPCR from both tissue and bacteria obtained in the remaining culture media. The serial passages into new rabbits were done inoculating *TPA* from culture media into their testis for mass propagation.

#### *TPA* genomic sequencing and phylogenetic analysis

*TPA* enrichment and WGS was performed on extracted DNAs using the Illumina platform [24]. Samples with ≥40 copies of *polA*/μl were selected for WGS. DNA enrichment was performed using parallel, pooled whole-genome amplification (ppWGA) and/or custom 120-nucleotide RNA oligonucleotide baits (Agilent Technologies, Santa Clara, CA, USA; SureSelect XT Low Input). Pooled, *TPA*-enriched libraries were then sequenced using the MiSeq platform (Illumina, San Diego, CA, USA) at UNC. Sequencing data were processed using an adaptation of our previously described bioinformatics pipeline (see https://github.com/IDEELResearch/Tpallidum_genomics). Briefly, sequencing data were subjected to quality control using fastqc (0.11.9) [34], adapter removal using Trimmomatic (0.36) [35], and host genome removal using BBMap (38.82) [36]. Reads were then aligned using BWA (0.7.17) [37] to a custom Nichols reference genome (CP004010.2) and variant calling was performed using GATK (3.8.1) [38]. Following variant calling, SNP site was used for phylogenetic analysis. Multiple sequence alignment was performed using MAFFT (v7.490) [39]. Putative recombination regions were masked using Gubbins (v3.2) [40] and maximum likelihood (ML) tree was constructed using RAxML (v8ꞏ2ꞏ12) [41] with GTRGAMMA substitution model and 1,000 rapid bootstraps. Trees were visualized and annotated in R (v4.1.2) using the ggtree package (v3.2.1), and clades were assigned. *TPA* population assignments previously made using Bayesian modeling for a subset of samples compared to the larger global *TPA* population, as recently described [25], were annotated to facilitate comparison to published literature. Macrolide resistant strains were identified using competitive mapping of ribosomal 23S gene as previously described [42, 43]. Raw sequencing data from this study with residual human reads removed are available through the Sequence Read Archive (SRA, BioProject PRJNA815321).

#### Structural mapping of *T. pallidum* BamA

*bamA* (*tp0326*) sequences were extracted from the genomes for mapping onto a three-dimensional model obtained from the AlphaFold database (AF-O83346-F1) [44]. Sequence alignments were visualized and identified mutations were mapped onto the protein model using UCSF Chimera 1.1 [45].

#### Statistical analysis

The databases from the prior studies were reviewed, merged and exported by the Epidemiology and Biostatistics Unit of CIDEIM on September 22^nd^ of 2022. The retrospective database used consistent variable names with the prospective database in REDCap to facilitate the analysis. The merged final database was imported in Stata 16.0 (Stata Corp, Stata Statistical software: Release 16, 2019) for analysis. A descriptive analysis was conducted to identify absolute and relative frequencies of categorical variables and median and range of quantitative variables. The Fisher exact test was used to compare *TPA polA* positivity by sample type and DFM with qPCR.

#### Ethics statements

The prospective enrollment of participants with ES in prior studies between 2014 and 2019 was reviewed, approved and monitored by CIDEIM IRB (protocol reference numbers 1204, 1264 and 1281). The retrospective inclusion of samples and data from prior studies in this analysis was approved by CIDEIM IRB (protocol reference number 1315) as well as the prospective enrollment of participants after 2019 (protocol reference number 1289). The ethics committees, and scientific directors of the referring health institutions in Cali, also approved the protocols. The IRB of the University of North Carolina approved the master protocols (approval codes 19–0311 and 21–3181). All clinical research activities were conducted in accordance with NIH good clinical practice standards and the Declaration of Helsinki guidelines. Written informed consent was obtained prior to enrollment from all participants. All patients with a diagnosis of ES evaluated at CIDEIM received benzathine penicillin treatment according to centers for disease control (CDC) sexually transmitted infections treatment guidelines [46], regardless of their decision to ultimately participate in the study. Treatment was offered without charge for sexual contacts voluntarily identified by the study participant. Participants’ transportation costs were covered by the study regardless of their decision to participate.

Protocols for animal use were reviewed and approved by the Institutional Ethics Committee for the Care and Use of Animals in Experimentation of ICESI University (reference 0021/2019). The study was conducted according to Law 84 of 1989 of the Republic of Colombia and university statute N° 847 (9th of July of 2012). Animal maintenance and husbandry was conducted following the Guide for the Care and Use of Laboratory Animals (8th Edition). Research and animal facility staff were trained on the study procedures, including the intratesticular inoculation procedure, and followed detailed standard operative procedures developed at UConn Health. When required, procedures were carried out under intramuscular sedation (Tranquilan and Xilasyne) and intravenous anesthesia (Tiletamine–Zolazepam), with meticulous attention to minimizing any potential suffering endured by the animals.

## Results

### Three different surveillance methods for recruitment of individuals with early syphilis

Using the active surveillance systems described above, we identified a total of 1,966 participants with possible ES over an eight-year period (2014–2022). Non-eligible participants where either asymptomatic at the time of repeat clinical evaluation at allied health institutions, had already received antibiotic treatment for syphilis or denied having signs and symptoms of ES during phone call interviews, and, in some cases, could not be reached. Of the 134 (7%) participants invited for an in-person clinical evaluation at CIDEIM following initial screening, 128 agreed to participate and were enrolled (**Fig 1**). Of the 128 participants, 52 (40.6%) were retrospectively included in this analysis and 76 (59.4%) were prospectively recruited. Participant recruitment using syphilis testing laboratory databases identified the largest number of individuals (n = 52), followed by targeted subject identification at network health centers (n = 43) and routine syphilis screening of PLWH in two private HIV clinics (n = 33). However, the proportion of patients enrolled was highest from our public sector health center network (76%), followed by enrollment in two HIV clinics (13%), and, lastly, by the syphilis laboratory database surveillance strategy (3%). ES participants were identified in 21 of the 22 Cali city districts and from five neighboring municipalities over an 8-year period. The three districts with the highest number of cases are heavily populated with socioeconomically disadvantaged individuals.

### Sociodemographic characteristics and STI risk factors associated with early syphilis

**Table 1** presents a detailed distribution of sociodemographic features and risk factors for ES in our patient cohort. Our cohort consisted of a young population with a mean age of 30 years. Most patients (52%) self-identified as heterosexual; 38% were MSM and 10% bisexual. Two-thirds were cis-gender men, 63% patients were of mixed ethnic background, and 72% were unmarried. More than half (59%) had attended high school, 11% just elementary school, and 2% had no formal education. Of particular interest, 7% of cases were in men and women who recently had immigrated illegally from neighboring Venezuela because of recent sociopolitical instability in that country. Most patients (94%) had their first sexual encounter before the age of 20, many (45%) before the age of 15. A large proportion of participants (64%) reported having two or more sexual partners within 12 months prior to enrollment, while 18% self-reported more than 5 sexual partners. Several patients gave a history of sexual abuse during childhood, including one subject who claimed they were sexually assaulted at the age of 9. Commercial sex work was self-identified as a risk factor in 18 participants. Within this group, five participants (27%) were cis-gender MSM, four (22%) were bisexual / cis-gender men, and one (5%) subject self-identified as a transgender woman.

**Table 1:** Sociodemographic and sexual behavior characteristics among enrolled participants.

	Primary syphilis (n = 15) (%)	Secondary syphilis (n = 112) (%)	Total (n = 127) (%)
**Mean age (SD)**	28.2 (+/-8.8)	30.5 (+/-10.8)	30.2 (+/-10.5)
**Migrant [n (%)]**			
• **No**	13 (86)	105 (93.7)	118 (93)
• **Yes**	2 (14)	7 (6.3)	9 (7)
**Gender [n (%)]**			
• **Trans-women**	0	1 (1)	1 (0.7)
• **Cis women**	3 (20)	39 (35)	42 (33)
• **Cis men**	12 (80)	72 (64)	84 (66.3)
**Sexual orientation [n (%)]**			
• **Bisexual**	3 (20)	10 (9)	13 (10)
• **Heterosexual**	7 (47)	59 (53)	66 (52)
• **MSM**	5 (33)	43(38)	48 (38)
**Race [n (%)]**			
• **Indigenous**	0	1 (0.8)	1 (0.7)
• **White**	1(7)	10 (9)	11 (9)
• **Afro-Colombian**	1(7)	34 (30)	35 (28)
• **Mixed race**	13(85)	67 (60)	80 (63)
**Educational status [n (%)]**			
• **No formal education**	0 (0)	2 (1.8)	2 (1.5)
• **Primary school**	3 (20)	11 (10)	14 (11)
• **High school**	8 (53)	67 (60)	75 (59)
• **Trade school**	2 (13)	23 (20.2)	25 (20)
• **Undergraduate/Graduate**	2 (13)	9 (8)	11 (8.5)
**Marital status [n (%)]**			
• **Divorced**	0	4 (4)	4 (3)
• **Single**	10 (67)	81 (72)	91 (72)
• **Cohabitating/ Married**	5 (33)	27 (24)	32(25)
**Age of first sexual encounter [n(%)]**			
• **< 15 years**	6 (40)	39 (35)	45 (35)
• **15–19 years**	8 (53)	69(62)	77 (61)
• **> 19 years**	1 (7)	4 (4)	5 (4)
**Sexual partners in last year**			
• **0–1**	6 (40)	39 (35)	45 (35)
• **2–5**	9 (60)	49 (44)	58 (46)
• **> 5**	0	23 (20)	23 (18)
• **No data**	0	1 (0.8)	1 (0.7)
**Commercial sex [n (%)]**	0	18 (16)	18 (14)

### Clinical and laboratory findings

**Fig 2:**
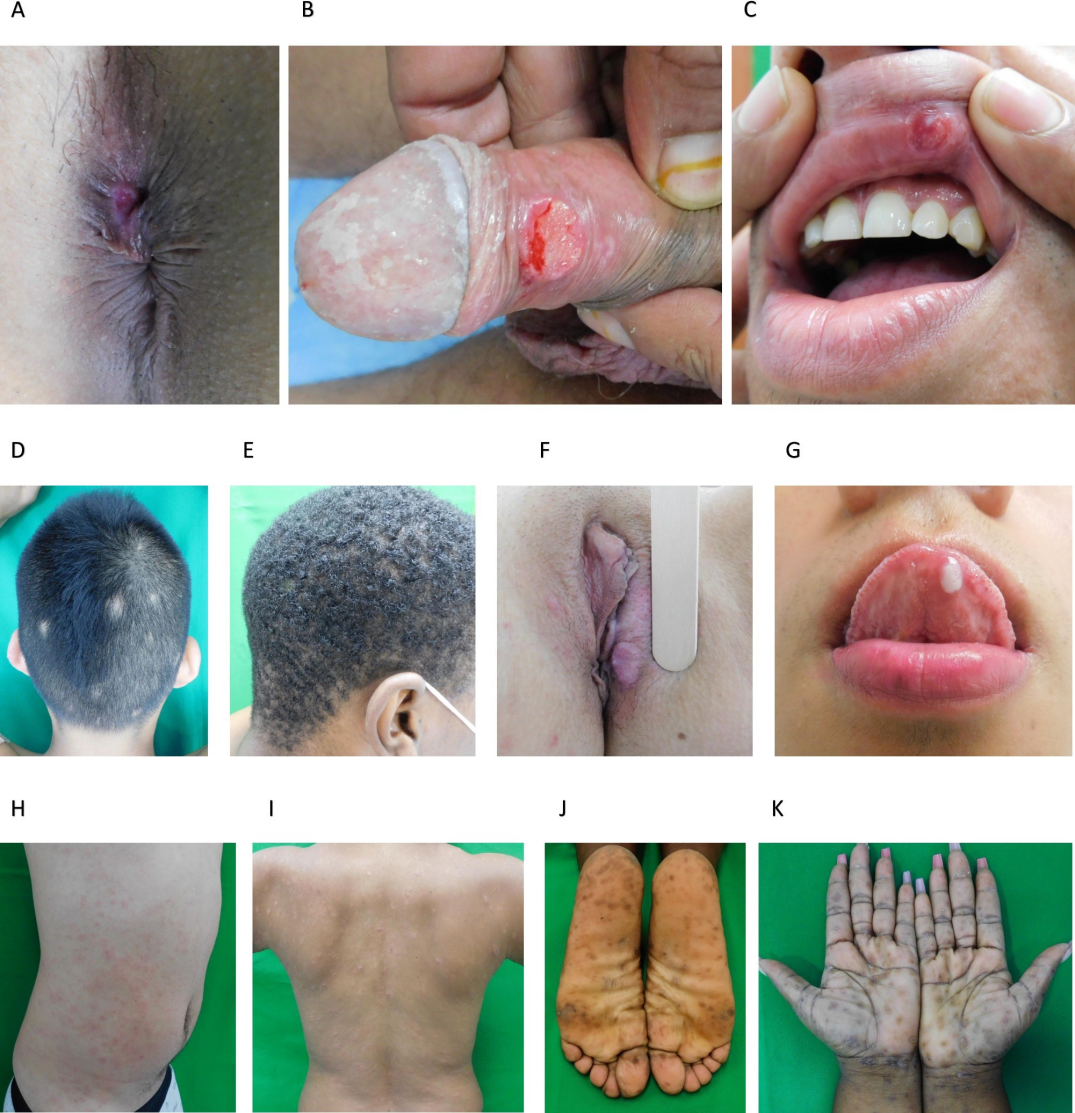
Clinical features of early syphilis in our patient cohort. **(A-C)** Chancres. **(D and E)** Patchy “moth eaten” alopecia of secondary syphilis. **(F)** Condyloma lata. **(G)** Mucous patches of secondary syphilis. **(H-I)** Diffuse maculopapular exanthems of secondary syphilis. **(J-K)** Classic palmar and plantar rashes of secondary syphilis.

Of the 128 participants enrolled, 15 (12%) had PS, 112 (87%) had SS, and 1 (1%) ELS (**See Table 2**). All patients, except one diagnosed with PS, had reactive RPRs and positive confirmatory TTs. The only PS patient with a nonreactive RPR and negative rapid TT, had a genital ulcer swab positive for *TPA polA* DNA. All PS patients had characteristic oral or genital ulcers (**Fig 2A–2C**), in some cases, multiple ulcers affecting the penis and perianal regions. One third of patients with PS were co-infected with HIV, three (20%) had a previous history of syphilis, and one was pregnant at the time of diagnosis. All SS participants had characteristic exanthems, most commonly a macular rash predominantly affecting the trunk, upper and lower extremities, palms, and soles (**Fig 2H–2K**). 33% of SS patients had generalized lymphadenopathy, 17% had mucous patches, 10% had condyloma lata, and 9% had patchy alopecia **(Table 2** and **Fig 2D–2G)**. Within the SS group, 44 (39%) were HIV positive, 8 (7%) were co-infected with either hepatitis B or hepatitis C, and almost a third had a previous episode of syphilis. Four women diagnosed with SS were pregnant at the time of enrollment; three recently had migrated from Venezuela and had limited access to adequate prenatal care. All four pregnant women were treated with benzathine penicillin.

**Table 2:** Clinical characteristics among enrolled participants with early syphilis.

	Primary syphilis n = 15 (%)	Secondary syphilis n = 112 (%)	Total n = 127 (%)
**Pregnancy [n (%)]**	1 (7)	4 (3.5)	5 (4)
**PLWH [n (%)]**	5 (33)	44 (39)	49 (38)
**Previous history of syphilis**	3(20)	32 (28)	35(27)
**Hepatitis B co-infection**	0	8 (7)	8 (6)
**Hepatitis C co-infection**	0	8 (7)	8 (6)
**Number of genital ulcers**			
• **1 ulcer**	8 (53)	/	8 (4)
• **≥ 2**	7 (47)	/	7 (5)
**Location genital ulcers**			
• **Vulva**	3 (20)	/	3 (2)
• **Penis**	9 (60)	/	9(7)
• **Penis/Mouth**	2 (13)	/	2 (2)
• Penis/Anal*	1 (6)	/	1 (1)
Rash*		112 (100)	112 (88)
• **Nodular**	/	1 (1)	1 (1)
• **Papular**	/	1 (1)	1(1)
• **Papular-scaly**	/	1 (1)	1 (1)
• **Psoriasis-like**	/	2 (2)	2 (2)
• **Maculo-papular**	/	36 (32)	36 (28)
• **Macular**	/	71 (63)	71 (56)
Rash location*			
• **Face/head**	/	5 (4)	5 (3.9)
• **Genitals**	/	22(20)	22(17)
• **Limbs**	/	75 (67)	75 (60)
• **Palms and/or soles**	/	87(78)	87(68)
• **Trunk**	/	99(88)	99(78)
Lymphadenopathy*	/	42 (37)	42 (33)
Condyloma lata*	/	13 (12)	13 (10)
Mucous patches*	/	22 (20)	22 (17)
Patchy alopecia*	/	11 (9.8)	11 (9)

PLWH–people living with HIV

*Non-exclusive data

### *T. pallidum* burdens in whole blood, skin, and mucosal lesions

We detected *TPA* DNA in 73% (93/127) of all patients studied (**Table 3**). Several patients had more than one PCR-positive sample. 73% of PS genital ulcer swabs were PCR-positive with a mean *polA* copy number of 67 copies/μl. 62% of SS skin lesions were PCR positive with an average of 42 copies of *polA*/μl. As expected, WB *polA* positivity was more common in SS (44%) than PS (7%) patients (p-value = 0.009). DFM proved to be a useful tool for the rapid identification of spirochetes in genital ulcers but was not as sensitive as molecular diagnosis; 73% of PS ulcers were positive by PCR as compared to 46% by DFM (**Table 4**). All lesions positive by DFM also were positive by PCR, whereas three PCR-positive ulcers were DFM negative.

**Table 3:** Median *TPA polA* copies and % positive by syphilis stage and sample type.

	Number of samples	Positive* [n (%)]	Median *polA* copies/μl
**Primary syphilis (PS) [n = 15]**			
**Genital ulcer swabs**	15	11 (73)	67 (10–8,835)
**Whole Blood**	14	1 (7)	N/A
**Secondary syphilis (SS) [n = 112]**			
**Condyloma lata swabs**	34	21 (61)	36 (9–1,361)
**Skin biopsies**	101	63 (62)	42 (6–455)
**Whole Blood**	110	46 (42)	2 (2–6)

* Individual patient sample with highest *polA* burden was selected for calculating median

**Table 4:** Correlation between darkfield microscopy (DFM) results and *TPA polA PCR* % positive in matching genital ulcer swabs.

	*polA* PCR (-)	*polA* PCR (+)	Total
**DFM (-)**	3 (43%)	4 (57%)	7 (54%)
**DFM (+)**	0 (0)	6 (100%)	6 **(46%)**
**Total**	3 (23%)	10 **(77%)**	13

### Rabbit Infectivity Testing (RIT)

As noted in previous studies [12, 14], *polA* copy numbers in blood often are too low for detection by qPCR. RIT on the other hand is highly sensitive [47] and enables isolation of large quantities of *TPA* DNA from isolated, rabbit passaged organisms. To implement RIT in Cali, Colombia ‐ where year-round warm temperatures made it difficult for our animal facility to reliably maintain temperatures at or below 12°C ‐ we designed a novel refrigerated housing cabinet. This cabinet houses four individual rabbits in a comfortable, safe, and climate adjusted environment. A total of 30 rabbits were used for this study. At the outset, two rabbits were used to confirm that the climate conditions within the cabinet were conducive to successful intratesticular propagation of *TPA*. A total of nine rabbits were inoculated with patient specimens for the RIT experiments (**Table 5**). Two rabbits were inoculated with samples from PS (one whole blood and one chancre exudate); seven were inoculated with WB from patients with SS. The rabbit inoculated with lesion exudate developed testicular necrosis and required euthanasia. Five of the eight rabbits inoculated with WB (1 PS and 4 SS) seroconverted (*i*.*e*., reactive rapid TT and NTT) within 90 days; from these, four isolates were obtained. Three of the four RIT positive WBs had very low *polA* copy numbers by qPCR, while the fourth was qPCR negative. All four RIT yielded sufficient numbers of spirochetes to conduct WGS and were further passaged to bank organisms for future investigation. An isolate was not recovered from one rabbit that seroconverted following inoculation with qPCR negative WB.

**Table 5:** Rabbit infectivity *polA* positivity compared to *TPA* DNA extracted from ES patient’s skin and blood.

Clinical Stage	Copies of *polA*/μl			Days to Seroconversion†	# of rabbit passages for WGS	Copies of *polA*/μl
	Skin lesion	Genital ulcer	Whole blood			Testicular extract following RIT
PS	NA	67.0	0.04	87	1st	29,947
PS + HIV^§^	NA	650.9	1.02	NA	NA	0
SS	1.1	NA	0	NR	NA	0
SS	174	NA	0.4	NR	NA	0
SS + HIV	15.2	0.34	0	37	1st	10.2^*^
SS + HIV	5.2	36.1	0	NR	NA	0
SS + HIV	18.2	52.5	0.8	74	2nd	775.0
SS + HIV	NA	14.5	0.29	85	2nd	4,574
SS + HIV	554	334.0	0	63	2nd	32,760^#^

NR–Nonreactive by day 90 post-inoculation; NA–not applicable

*–sequencing failed

#–yet to be sequenced

†–As determined by a reactive rapid TT and confirmatory NTT

§–The rabbit was injected with material from a chancre and developed testicular necrosis requiring euthanasia.

### Early syphilis patients’ *T. pallidum* whole genome sequencing

**Fig 3:**
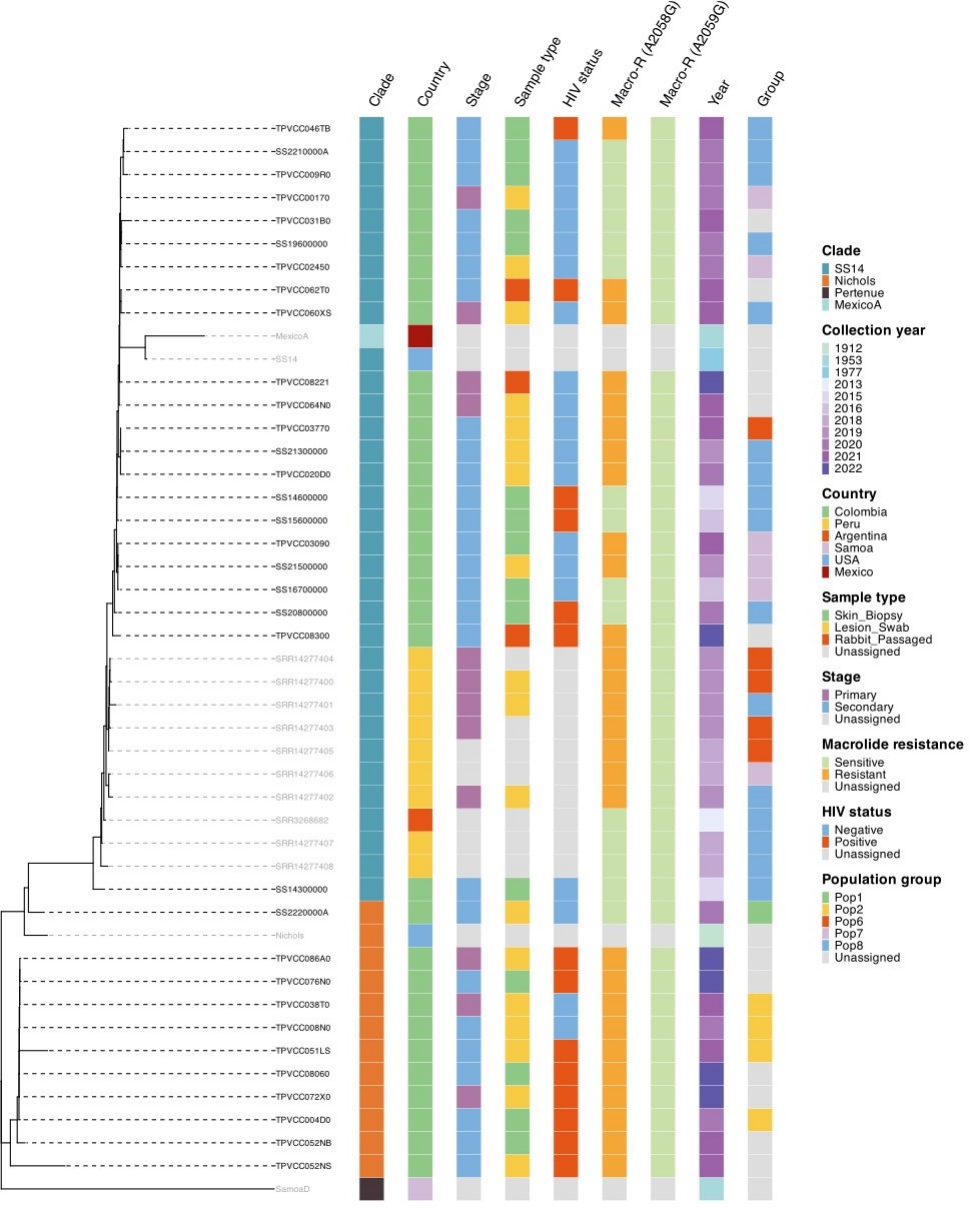
Recombination-masked *TPA* whole-genome phylogeny derived from 33 unique specimens obtained from 32 individuals enrolled in Cali, 10 recently published genomes from Peru and Argentina, and three reference *TPA* genomes (MexicoA, SS14, Nichols) and one *Treponema pallidum* subsp. *pertenue* reference genome (SamoaD). The presence of *TPA* 23S rRNA mutations associated with macrolide resistance (A2058G and A2059G) are shown. *TPA* population groups previously assigned using Bayesian modeling in a recent analysis by Seña *et al*. [25] are included to facilitate comparison to global *TPA* strains.

A major objective of the current study was to evaluate the diversity of *TPA* strains circulating in Cali. WGS was conducted on 33 ES Cali samples that had acceptable coverage for phylogenomic analysis, 24 of which were included in our recent analysis of *TPA* global genetic diversity [25]. In one case, we had more than one sample from the same patient. Publicly available *TPA* genomes from 10 ES patients from South America (9 from Peru and 1 from Argentina), three *TPA* reference genomes and one *Treponema pallidum* subsp. *pertenue* reference genome were included in the phylogenomic analysis. WGS revealed that SS14-lineage strains predominated (**Fig 3**). Among the subset of genomes included in a recent analysis of global *TPA* genetic population structure by Seña et al [25], we identified five distinct *TPA* populations, with the greatest number in the SS14-lineage (labeled in **Fig 3** as Populations 6–8). Within the Nichols-lineage, we found one predominant subpopulation (labeled in **Fig 3** as Population 2). Over 50% of all samples sequenced had at least one mutation associated with macrolide resistance.

### Sequence conservation of an immunodominant extracellular loop and opsonic target in BamA (TP0326)

**Fig 4:**
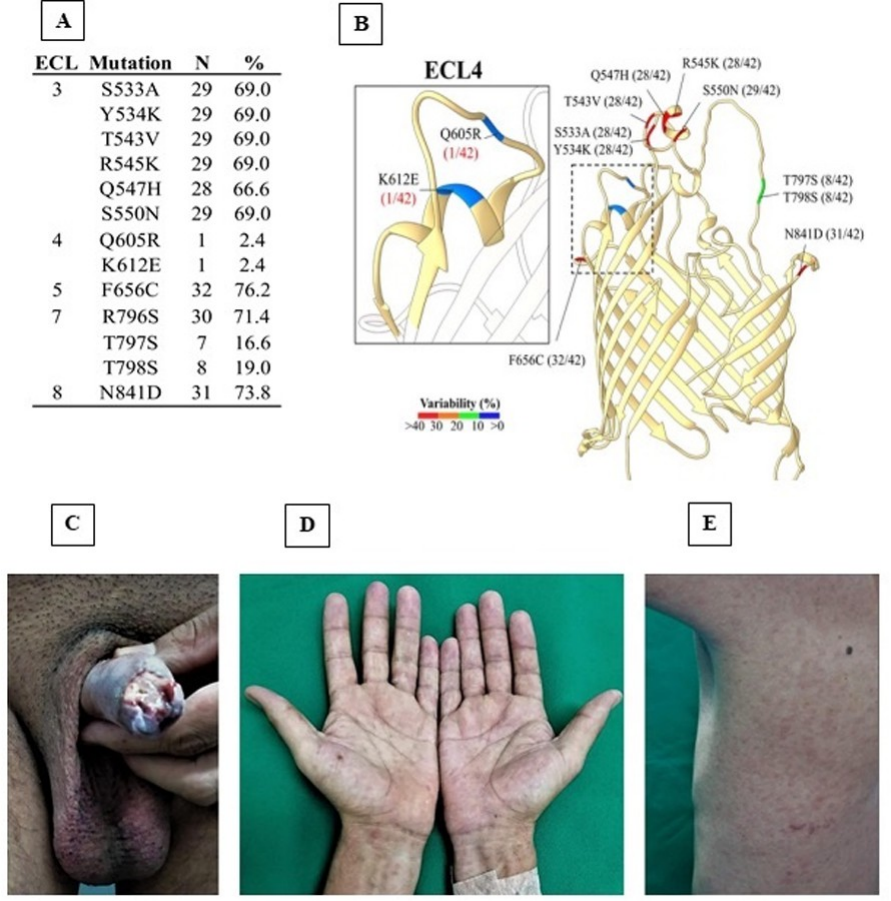
**(A)** Variability in the BamA β-barrel occurs predominantly in extracellular loops (ECL) 3, 5, 7, and 8. **(B)** Location in the BamA β-barrel of ECL variants shown in **Fig 4, panel A**. Colors indicate the frequency of each substitution. The blowup shows the two amino acid substitutions [glutamine to arginine (Q605R) and a lysine to glutamic acid (K612E)] in ECL4 of the BamA proteoform in *TPA* strain infecting early syphilis patient shown in panels C-E. POTRA domain not displayed to improve visual clarity of the BamA β-barrel domain. **(C-E)** Destructive penile genital ulcer, macular rash on palms and wrists and roseola like exanthem over the torso in the patient with ECL 4 mutations.

The syphilis spirochete’s OMPs are considered the most promising candidates for vaccine development. [27, 30, 48, 49] The spirochete’s repertoire of OMPs includes BamA (β- barrel assembly machinery subunit A ‐ TP0326), the central component of the molecular machine that inserts newly exported OMPs into the outer membrane. [29, 50] BamA is bipartite, consisting of a 16-stranded β-barrel with eight ECLs and a periplasmic arm containing five POTRA (polypeptide transport-associated) domains. [27, 29] We previously showed that ECL4 of BamA Nichols is an immunodominant ECL and that polyclonal and monoclonal antibodies directed against it promote opsonophagocytosis by rabbit peritoneal and murine bone marrow-derived macrophages, respectively. [48] We also noted that some *TPA* strains in Cali contain an amino acid substitution in BamA ECL4 that markedly affects its reactivity with the antibodies in syphilitic sera directed against Nichols BamA ECL4. [29, 30] Consequently, it was of interest to use our genomic sequences to assess the variability of BamA, particularly ECL4, in South American strains (n = 32 from Cali; n = 9 from Peru; n = 1 from Argentina). Of note, the *ab initio* model predicted by AlphaFold used herein contains a longer ECL3 than the prior model generated by ModWeb based on the *Neisseria meningitidis* BamA ortholog. [27, 29] As shown in **Fig 4A**, ECL3, 5, 7, and 8 are highly variant in South American *TPA* strains. Contrary to expectations, we found only one Cali *TPA* strain of the 42 *TPA* strains analyzed contained mutations in ECL4 (**Fig 4B**); these give rise to two nonconservative amino acid substitutions (Q605R and K612E). *TPA* DNA for this strain was acquired from an HIV-negative subject at the interface between PS and SS who presented with a severely inflamed penile ulcer (**Fig 4C**), palmar lesions (**Fig 4D**), and exanthem over his trunk and abdomen (**Fig 4E**). He responded well to penicillin therapy, exhibiting full resolution of symptoms and complete healing of the genital ulcer.

## Discussion

This study characterizes the clinical, epidemiologic, and complex sociodemographic features of a cohort of participants diagnosed with venereal syphilis in a large urban center in Colombia. Our findings underscore the importance of active surveillance and timely diagnosis to avoid clinical complications and diminish transmission of the bacterium. As in prior studies [12–16], we show that syphilis continues to be a serious public health problem in Colombia, including high-risk groups, such as commercial sex workers, MSM and PLWH. Our findings also illustrate the importance for vaccine development of identifying regional sequence variants of candidate OMP vaccinogens.

Although health care services by the Cali public health network are free, many of our participants had several days delay in diagnosis and treatment of ES. Of particular concern, our study team determined that public health network laboratories often wait for the patient to voluntarily claim syphilis serology results rather than routinely informing their provider of reactive serologies. This approach can result in a significant delay in treatment. In addition, treatment for sexual partners remains challenging, given that partner notification in the city is not standardized and sexual partners may be very difficult to track and contact. A new risk factor was uncovered in our study, in that 7% of our participants, including one pregnant woman, were recent migrants from Venezuela. Individuals seeking asylum, as in this case, are very likely to experience difficulties obtaining necessary health care. In this study, immigrants often reported to be engaged in commercial sex trade for financial survival. Health care authorities must prioritize early identification and treatment for this underserved population.

The clinical and laboratory findings in the present study, as in our own published work [12, 14, 51], underscore the systemic and relapsing chronic inflammatory and infectious nature of ES. Indeed, most of our ES patients presented with persistent and disseminated dermal and cutaneous lesions, all had constitutional symptoms, and many had lymphadenopathy. *TPA* DNA was extracted from at least one site (blood, mucous membranes and/or skin) in 73% of all ES patients studied. Genital ulcers from PS patients had the highest yield (73%), as well as the highest copy numbers, including one patient with PS that had over 10,000 copies of *polA* in a genital ulcer swab. In line with published reports [52], spirochetemia, determined by qPCR, was much more common in SS patients (42%) than in PS patients (7%). Our temperature-controlled cabinet for housing rabbits proved to be very useful for isolating *TPA* strains and obtaining yields of *TPA* DNA amenable for WGS. This cabinet, which employs a mobile cooling unit, can be deployed in clinical sites located in regions with tropical climates. While *TPA* DNA alone is sufficient to establish OMP sequence diversity, live strains can be used for experimentation, including vaccine trials in rabbits.

Until now, little was known about the genetic diversity of *TPA* in Colombia and similar countries in South America. Predominance of SS14-lineage strains in this region of the world (**Fig 3**), as in other locations [24, 25, 43], is likely the result of recent global clonal expansion of this clade. SS14-lineage samples had less genetic variation than Nichols-lineage samples, and clustered with three sub-populations, in comparison to two subpopulations in Nichols. Unfortunately, neither recombination-masked phylogenomic analysis nor conventional molecular typing are sufficient to inform vaccine development because they often exclude genes encoding key surface antigens a prospective vaccine would target. To be used for vaccine development, genomic analysis needs to focus on sequence variability of the surface antigens used to generate a protective immune response. [27, 28] Along these lines, we analyzed the β-barrel domains of BamA (TP0326). Although a larger study is needed, our findings suggest that ECL4, a proven opsonic target [27, 29, 31], is mostly conserved, a highly desirable property for a vaccine antigen.

The study has several limitations that must be taken into consideration. Firstly, because patients were enrolled from several cohorts and over an eight-year period, the full extent of OMP and ECL variability will require additional testing in a larger cohort. Second, we first focused on secondary syphilis patients and over the past few years on all patients with early syphilis. As a result, we cannot be certain that the molecular epidemiology of primary syphilis in Cali is fully reflected in this cohort. Lastly, our patient population is exclusively from southwestern Colombia and may not represent the true magnitude of strain diversity and ECL mutations throughout the country. A broader survey that includes additional locations in Colombia as well as other Latin American countries will be needed to optimally inform syphilis vaccine development.

In summation, our study design and findings underscore the importance of promoting regional development for future syphilis vaccine trails. Such infrastructure allows for improved diagnostics and facilitates training of health care personnel in the proper diagnosis of syphilis and other STIs. It also engenders a sustainable partnership between clinician scientists, epidemiologists, and local and central governments. Our findings also underscore that development of a vaccine with global efficacy depends on determination of regionally prevalent sequence profiles of OMPs and specific ECLs targeted by a vaccine.
